# Parametric Studies of Polyacrylamide Adsorption on Calcite Using Molecular Dynamics Simulation

**DOI:** 10.3390/molecules30020285

**Published:** 2025-01-13

**Authors:** Keat Yung Hue, Jin Hau Lew, Omar K. Matar, Paul F. Luckham, Erich A. Müller

**Affiliations:** Department of Chemical Engineering, Imperial College London, London SW7 2AZ, UK; v.hue20@imperial.ac.uk (K.Y.H.); s.lew20@imperial.ac.uk (J.H.L.); o.matar@imperial.ac.uk (O.K.M.); p.luckham01@imperial.ac.uk (P.F.L.)

**Keywords:** adsorption, molecular simulation, parametric studies, surfaces, polymers

## Abstract

This study investigates the efficacy of polyacrylamide-based polymers, specifically hydrolysed polyacrylamide (HPAM), in reducing solids production within carbonate reservoirs. Building on our earlier simulation approach, molecular simulations were conducted to examine how these polymers adsorb onto calcite, the main mineral found in carbonate formations. The adsorption process was affected by several factors, including polymer molecular weight, charge density, temperature, and salinity. Generally, increased molecular weight, charge density, and temperature resulted in higher adsorption rates. The effect of salinity was more nuanced, as salt-bridging and charge-screening effects created competing influences. The simulation outcomes correspond closely with experimental results, offering valuable insights for designing and optimizing polymer-based strategies aimed at controlling solids production in carbonate reservoirs.

## 1. Introduction

In the energy industry, the term “formation strength” refers to the mechanical properties of reservoir rocks to withstand the stresses exerted upon them. Solids production becomes an issue in weak and poorly consolidated carbonate formations when the induced formation stress surpasses the formation strength [1,2,3]. This can lead to the detachment of solid particles which are then transported with the reservoir fluids, potentially wearing surface equipment and increasing environmental waste [4]. The solids production risk can be reduced by injecting formation-strengthening chemicals, where these chemicals have been investigated experimentally [5]. However, screening suitable chemicals can be time-consuming and experimentally challenging. Molecular simulation offers an alternative approach to expedite chemical-screening processes by suggesting potential candidates to complement experimental synthesis. The performance of these chemicals can be evaluated in terms of their molecular conformation and adsorption characteristics onto calcite surfaces, which is the main component of carbonate formations.

In our previous manuscript [6], various polyacrylamide-based (PAM) polymers, including basic PAM, hydrolysed polyacrylamide (HPAM), and sulfonated polyacrylamide (SPAM), were evaluated using adsorption free energy analysis at room temperature and in vacuum conditions, with HPAM showing the best adsorption performance. However, polymer adsorption may be influenced by more challenging reservoir environments characterised by higher pressure, temperature, and salinity. Additionally, polymer characteristics such as molecular weight and charge density have been shown to impact polymer adsorption mechanisms. Understanding the effects of these parameters on polymer–calcite interactions is essential for predicting polymer adsorption performance to complement experimental studies.

It is also important to note that polymers are typically injected as solutions during formation consolidation, requiring parametric studies to be conducted in aqueous environments. Nevertheless, previous work has shown that in water solvents, the highly hydrophilic PAM-based polymers result in nearly zero free energy value, complicating the analysis of polymer performance across different parametric studies. This manuscript will deploy the equilibrium adsorption simulation approach, focusing on polymer adsorption density on calcite surfaces in aqueous environments under varying polymer chemistries and reservoir conditions.

### Literature Review of Adsorption Parametric Studies

Extensive experimental and simulation studies have been conducted to investigate the effects of various parameters on polymer dynamics and their adsorption behaviours on mineral surfaces. These parameters include polymer molecular weight [7,8,9,10,11,12,13], charge density [7,8,12,14], functional group [13,15,16,17,18,19,20,21,22], pH [12,23,24], temperature [11,17,25,26,27,28], pressure [11,17], and salinity conditions [10,11,17,18,23,25,29,30,31,32,33,34,35]. Although not limited to formation-strengthening applications, MD simulations of PAM-based polymers have been predominantly reported for wastewater treatment and enhanced oil recovery (EOR) applications. This can be attributed to their excellent viscosifying and flocculating properties and their capability to form crosslinked hydrogels [36,37,38].

Abdel-Azeim and Kanj [11] conducted parametric studies on the dynamic and aggregation behaviour of HPAM polymer in aqueous solution. They found that increasing temperature and pressure did not significantly affect the polymer’s radius of gyration, $R_{g}$, and end-to-end distance, $R_{e}$. While increasing the polymer’s molecular weight results in a higher $R_{g}$, there is no consistent trend in polymer conformation, which can range diversely from coiled to extended shapes. In salt-free conditions, the polymer adopts an extended conformation due to electrostatic repulsion among the negatively charged functional groups. In contrast, a high salinity environment neutralises these charges, leading to a transition to a coiled conformation with reduced $R_{g}$. Chen et al. and Quezada et al. reported similar effects of salinity on PAM polymer conformational behaviour [31], with Quezada et al. concluding that this impact strongly correlated with polymer charge density. In their study of polymers, including PAM, HPAM, and SPAM in high-salinity solutions, Quezada et al. found that HPAM coiling was the most intense, followed by SPAM and PAM. This is because PAM is a neutral molecule, and SPAM has a lower charge strength and ion adsorption capacity compared to HPAM. Consequently, HPAM exhibits a lower $R_{g}$, potentially limiting its effectiveness in forming polymeric bridges for particle flocculation under high-salinity conditions.

The literature on adsorption parametric studies of PAM-based polymers on calcite surfaces is sparse. Ahsani et al. [28] employed both experimental studies and MD simulations to investigate the wettability alteration behaviour of HPAM on calcite surfaces. While calcite surfaces have an ionic structure and are inherently water-wet, natural carbonate formations are often surrounded by hydrocarbons with negatively charged carboxylates, which anchor to the surface and render them oil-wet. Their experiments recreated this calcite surface condition with pre-adsorbed oil components containing carboxylates. They found that HPAM could form a polymeric layer with surfactant-like behaviours to interact with the carboxylates or competitively adsorb to the surface and desorb the carboxylates, thereby modifying the calcite surface to a more water-wet state. A simpler model was used in their MD simulation without considering the pre-adsorbed carboxylates. The contact angle of water indicated HPAM adsorption based on the surface tension of the water–polymer–calcite phases. They observed that HPAM adsorption improved with increasing temperature from 20 °C to 80 °C in experiments, but a contradictory behaviour was noted in simulations. This disagreement in simulation results is attributed to increased polymer desorption with increasing kinetic energy. Another possible reason for the discrepancy could be the calcite crystal plane used in the simulations, where the surface consisted only of the carbonate (CO_3_^2−^) layer, resulting in stronger electrostatic repulsion with HPAM.

While not focusing on PAM polymers, Chen et al. [29] studied the interactions of a carbohydrate molecule, glucopyranose, with different calcite crystal planes. They observed that the adsorption of the carbohydrate decreased with increasing brine concentration across all crystal planes. Since the carbohydrate is a neutral molecule, the primary mechanism of calcite–carbohydrate interaction is governed by hydrogen bonding, with the crystal plane having the highest oxygen density showing the strongest affinity to the hydrogen atoms from the carbohydrates. Increasing brine concentration contributes to the formation of salt layers on the calcite surface, which screens the hydrogen bonding between calcite and carbohydrates and inhibits adsorption. They inferred that charged polymers typically used in EOR applications in high-salinity environments might follow a different mechanism. The charged functional groups of these polymers can have favourable electrostatic interactions with the adsorbed cations, resulting in increased polymer retention via salt bridging. Thus, they suggested that alternative non-ionic polymers could reduce polymer damage and retention on calcite surfaces.

Meanwhile, there are more simulation studies on the adsorption of PAM-based polymers on montmorillonite and quartz, both containing negatively charged surfaces. Ma et al. [16] investigated PAM-based polymers with different ionicities and found that cationic PAM had the most substantial adsorption to montmorillonite, followed by neutral and anionic PAM. This can be attributed to the electrostatic attraction from the cationic functional group, N(CH_3_)^3+^, and hydrogen bonding from the amide functional group, CONH_2_. A similar effect of polymer ionicity was reported by Qu et al. [17], who investigated different types of polymers, including neutral PAM. Additionally, they observed that higher pressure had a negligible effect on adsorption, and higher temperature reduced the adsorption of all polymers except PAM, whose adsorption remained stable at 300 K and 400 K with slight desorption at 500 K. Interestingly, despite the negatively charged surface, increasing salinity can improve the adsorption of both cationic and anionic polymers with negligible influence on neutral polymers. Sun et al. [35] investigated the effect of cation valency and found that divalent Ca^2+^ ions enhanced the adsorption of anionic PAM to montmorillonite more effectively than monovalent Na^+^ ions due to a stronger charge-screening effect and a reduction in electrostatic repulsion.

Regarding PAM adsorption on quartz surfaces, one of the most relevant studies for sand consolidation applications was conducted by Huang et al. [20]. They screened different types of potential chemicals and concluded that PAM exhibited the best adsorption performance. This performance was correlated to the strength of hydrogen bonding with the quartz surface, and the trend agreed with the reported experimental consolidation strength. In contrast, Yang et al.’s study [13] aimed to avoid polymer retention on quartz surfaces and assessed the performance of PAM-based polymers under different molecular weights and temperatures. They found that PAM adsorption increased with increasing molecular weight but decreased significantly with increasing temperature. Compared to Qu et al.’s study [17], the differing temperature trends of PAM adsorption on the montmorillonite surface suggest that interfacial properties may affect the adsorption mechanism. In another study, Quezada et al. [23,30] investigated the adsorption of HPAM on quartz under high-salinity conditions. They found that cations neutralised the electrostatic repulsion between HPAM and quartz, thereby improving adsorption. This finding is consistent with the results for the montmorillonite case.

To summarise, various parametric studies have been reported on the adsorption of PAM-based polymers on different surfaces. However, comprehensive research has not yet been explored to address the combined effects of reservoir conditions and polymer chemistry on adsorption to calcite surfaces, tailored explicitly for formation-strengthening applications. This gap underscores the need for further investigation. In this manuscript, we examine the influence of polymer molecular weight, charge density, temperature, pressure, and salinity on adsorption to calcite surfaces at typical reservoir conditions, with simulation results compared to experimental data whenever available.

## 2. Results and Discussion

### 2.1. Effect of Polymer Molecular Weight

The polymer adsorption, measured through both simulations and experiments at different molecular weights (MW), is depicted in Table 1, along with the simulated $R_{g}$ and $\kappa^{2}$. In simulations, polymer MW was represented in terms of repeat units (213 Da per repeat unit) as a surrogate for experimental candidates with varying MW. Despite experimental polymers having significantly higher MW, potentially reaching millions of Daltons, there was good agreement between simulations and experiments, with increasing polymer adsorption observed as molecular weight increased. This trend was expected because longer molecular chains experience stronger interactions with the calcite surface, increasing the contact area between the polymer and the surface and enhancing adsorption strength. In addition, since HPAM is a hydrophilic polymer, the shortest HPAM chains prefer to remain in the water and require a longer time to stabilise their adsorption onto the calcite surface.

While polymer MW positively impacts adsorption, both the experimental and simulation literature indicate that adsorption will reach an equilibrium value. Once the polymer adsorption reaches saturation, further increases in polymer MW or concentration do not enhance adsorption [7,8,39] and may even reduce stability [13]. This is attributed to changes in the adsorption mechanism with increasing polymer MW. Experimentally, it has been observed that polymer adsorption via polymer bridging is the strongest mechanism, followed by electrostatic bare patching [40]. At low MW, polymers primarily adopt a flat, extended conformation on the calcite surface, with electrostatic bare patching being the dominant adsorption mechanism. As MW or concentration increases, the polymer chains become compressed with a reduced effective contact area for each chain. This leads to the formation of more loops, with polymer bridging becoming the predominant adsorption mechanism [7,8,39]. Further increases in MW do not enhance adsorption due to the limited contact area and may inhibit existing polymer bridging between particles, potentially affecting adsorption stability.

In our simulation, given that only a single polymer chain was modelled and the calcite surface area was not saturated with polymer adsorption, the equilibrium adsorbed amount was not observed but was expected to be reached with higher polymer repeat units. Nevertheless, the polymer conformation with different MWs can be characterised by $\kappa^{2}$, as shown in Table 1. The increasing $R_{g}$ with higher MW was expected due to the larger polymer size. In contrast, the decrease in $\kappa^{2}$ with increasing MW indicates that the polymer transitions from an extended conformation to a coiled conformation, showing excellent agreement with theoretical expectations.

Since both the polymer adsorbed amount and $R_{g}$ increase with increasing MW, it is interesting to determine if a proportional relationship between them can be established in this study. We propose a simple relation to relate polymer $R_{g}$ with the polymer repeat unit. Polymer $R_{g}$ can be considered as the radius of the polymer globule, where the globular volume, V, can be approximated as the volume of a sphere:

(1) $$V\approx \frac{4}{3}\pi {R_{g}}^{3}$$

The total polymer mass, M, is directly proportional to the number of repeat units, N, and the mass of a single repeat unit, m:

(2) M=Nm

Assuming the polymer density, ρ, is constant, the polymer mass can be expressed in terms of V and ρ, approximated as shown in Equation (3). The number of repeat units thus has a proportional relationship with the $R_{g}$ as indicated in Equation (4).

(3) $$Nm=\rho V\approx \rho \frac{4}{3}\pi {R_{g}}^{3}$$

(4) $$\sqrt[{3}]{N}\propto \;R_{g}$$

**Figure 1:**
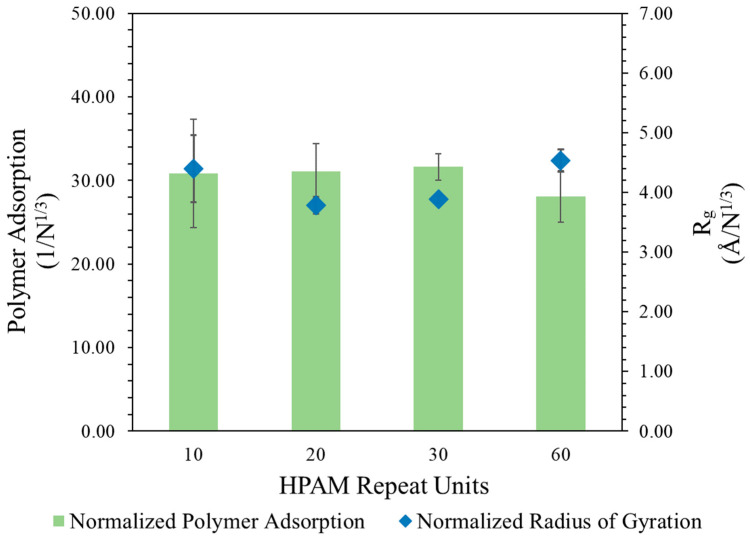
Polymer adsorption and radius of gyration, $R_{g}$, with normalization factor of cubic root of number of repeat units, $\sqrt[{3}]{N}$, at different molecular weights. Green bar indicates normalized polymer adsorption (left axis) and blue diamond symbol indicates normalized $R_{g}$ (right axis), with error bars representing standard error averaged over 5 realizations. Error bar of $R_{g}$ is significantly smaller, except for HPAM with 10 repeat units.

This proportionality was investigated by dividing the polymer $R_{g}$ and number of adsorbed atoms using the normalization factor $\sqrt[{3}]{N}$, with the results presented in Figure 1. To further validate this proportionality, another case of HPAM with 60 repeat units was also modelled in a larger water–calcite system to avoid system size effects. After normalization, it could be observed that the polymer $R_{g}$ and adsorbed amount reached a constant range with varying polymer repeat units, suggesting that their values increased proportionally with the cubic root of polymer repeat units during adsorption.

This implies that the $R_{g}$ of the polymer scales with MW at an exponent of 0.33 during adsorption to calcite surfaces, which diverges from the expected scaling relationship of greater than 0.5 typically seen in free solution under good solvent conditions [41]. This discrepancy can be attributed to the conformational changes that occur when HPAM interacts with the surface. Upon adsorption, the HPAM exhibits reduced flexibility, transitioning to a more coiled or globular conformation that is influenced by the increasing MW (as observed in $\kappa^{2}$ < 0.5, indicating nearly spherical shape). The observed lower exponent is consistent with findings in the literature, which suggest that globular polymers demonstrate a scaling behaviour characterized by lower exponents [41]. This indicates that the adsorption environment significantly alters the polymer conformation, resulting in a unique scaling relationship that highlights the complexities of polymer behaviour at interfaces.

### 2.2. Effect of Charge Density

Charge density (CD) is another polymer characteristic that can be fine-tuned to improve adsorption to calcite surfaces. The HPAM adsorption measured by simulations and experiments at different CDs and the polymer $R_{g}$s and $\kappa^{2}$s are detailed in Table 2. As CD increased, polymer adsorption improved with an agreeable trend in experiments and simulations. This improvement is attributed to the presence of more deprotonated carboxyl groups from the acrylate groups in the polymer (–COO^−^), which exhibit stronger attractive electrostatic interactions with the calcite surface.

Since all polymer models have similar repeat units and sizes, the trends in both $R_{g}$ and $\kappa^{2}$ are identical. The increase in both $R_{g}$ and $\kappa^{2}$ with increasing CD indicates that the polymer transitions to a more extended conformation during adsorption. This is due to the increasing electrostatic repulsions from the negatively charged functional groups in the chain, causing polymer segments to push away from each other and resulting in a more linear chain [42]. Meanwhile, a polymer with lower CD experiences less repulsion among the segments and prefers a coiled, more compact structure. The adsorption results reported here agree with the theory, with the literature suggesting that an optimum polymer CD should be employed as overly charged polymers can trigger charge reversal on the particle surfaces, inhibiting polymer bridging and adsorption [32,40].

### 2.3. Effect of Temperature

The results of polymer adsorption under the influence of temperature are interesting. As shown in Table 3, experimental data indicate that polymer adsorption deteriorates with increasing temperature, whereas simulations show an opposite trend. Theoretically, polymer adsorption is generally exothermic, and increasing temperature shifts the equilibrium state towards desorption [25]. This effect is further exacerbated by polymer degradation at high temperatures, causing the polymer to lose its effectiveness in bridging particles. The experimental results align with this theory, indicating that the adsorption effectiveness of HPAM could be inhibited by increasing temperature.

**Figure 2:**
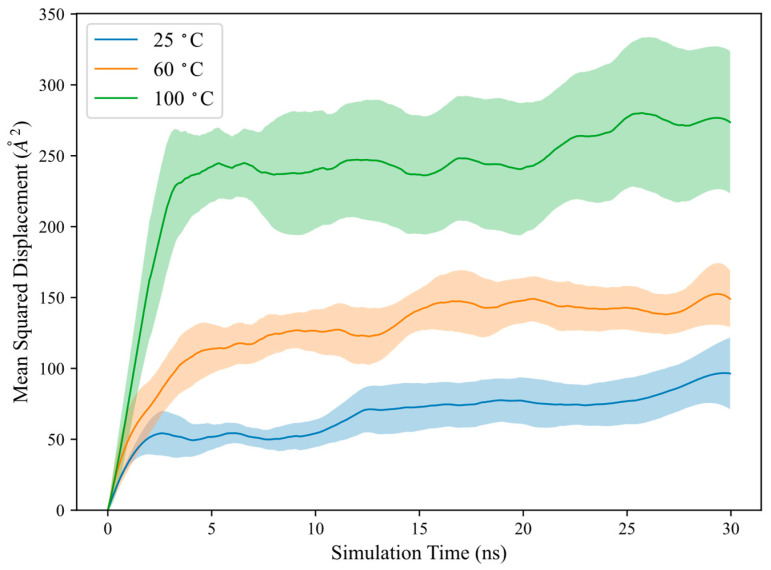
Polymer mean squared displacement at different temperatures. Shaded areas represent standard error from 5 realizations.

Meanwhile, the discrepancy in simulations with increasing polymer adsorption under higher temperature, though interesting, is not uncommon and has been reported in other studies. For example, a literature study on molecular adsorption on liposome surfaces observed rapid molecular transport kinetics at high temperatures with more negative adsorption free energy [43]. At higher temperatures, liposomes experience increased kinetic energy, which can influence adsorption behaviour depending on the enthalpic and entropic contributions to Gibbs free energy. Although adsorption typically results in decreased entropy due to reduced disorder, the author noticed a positive change in entropy. This is attributed to the replacement of water and counterions from the surface by adsorbate molecules, leading to an overall increase in entropy upon adsorption. To investigate the transport properties of HPAM, polymer mean squared displacement, MSD, and self-diffusion coefficients, D, are computed below [44]:

(5) $$MSD=\langle {\left[r_{i}(t)-r_{i}(0)\right]}^{2}\rangle$$

(6) $$D=\frac{1}{6}\underset{t\to \infty }{\lim}\frac{d}{dt}\langle {\left[r_{i}(t)-r_{i}(0)\right]}^{2}\rangle$$

where $r_{i}(0)$ is the coordinate at the initial time and $r_{i}(t)$ is the coordinate at different times. D can be measured from the slope fitted over the linear region of the MSD versus the time plot, as depicted in Figure 2 for polymer MSD at different temperatures. It is important to note that D is typically valid only for free polymers in solution. In this study, D is computed to characterise the polymer transport behaviour during adsorption and is measured over the linear slope at the initial timesteps. The computed D, along with polymer $R_{g}$ and $\kappa^{2}$, are presented in Table 4.

The results indicate that polymer MSD and D are significantly higher at elevated temperatures, suggesting that the polymer could diffuse more rapidly towards the calcite surface with increasing temperatures. The polymer could more vigorously explore its surrounding configurational space, leading to an increased contact area with the calcite surface and enhanced adsorption. This is further supported by the increase in $R_{g}$ and $\kappa^{2}$ with temperature, signifying that the polymer could adopt a more extended conformation to maximize adsorption contact with the calcite surface. Additionally, solvent quality may change with temperature, which could also influence the polymer–calcite interaction, although this is not considered in the current model.

In the literature, the influence of temperature on polymer adsorption has shown different trends in both experiments and simulations. Some studies report that polymers experienced enhanced adsorption with increasing temperatures [27,28,43,45], while others indicate increased desorption with higher temperatures [13,17,25]. The polymer–surface interactions can further complicate this inconsistency and require careful examination. In addition, a limitation of the simulation approach is its inability to account for polymer degradation at high temperatures. Our simulations do not consider structural changes in the polymer, which could be modelled only with a reactive forcefield. Consequently, the observed discrepancy between the simulation and experimental results may be attributed to the simulation focusing solely on thermodynamic factors without incorporating polymer degradation effects.

### 2.4. Effect of Pressure

As no experimental results are available for polymer adsorption under varying pressure, only simulation results will be discussed here. These results, including polymer adsorption, $R_{g}$, and $\kappa^{2}$ are summarised in Table 5. Increasing pressure showed no significant changes and led to only slight increases in polymer adsorption, $R_{g}$ and $\kappa^{2}$. This finding is consistent with the existing literature, which indicates that pressure has a negligible effect on adsorption and does not significantly impact polymer conformational behaviour [17]. This is expected, as the polymer solution remains in a liquid state and is less affected by pressure. Nevertheless, it has been reported that the rheological properties of PAM solutions, such as viscosity, can improve under extremely high pressures (above 200 bar) [46]. PAM exhibits noticeable shear-thickening behaviour at high-shear-stress deformations due to stronger intermolecular interactions and the formation of transient networks between polymer chains. While the enhanced viscosity of PAM under high pressure may strengthen polymer bridging, this effect is not captured in our adsorption study and requires further investigation to assess the impact of high pressure on PAM solution viscosity.

### 2.5. Effect of Salinity

**Figure 3:**
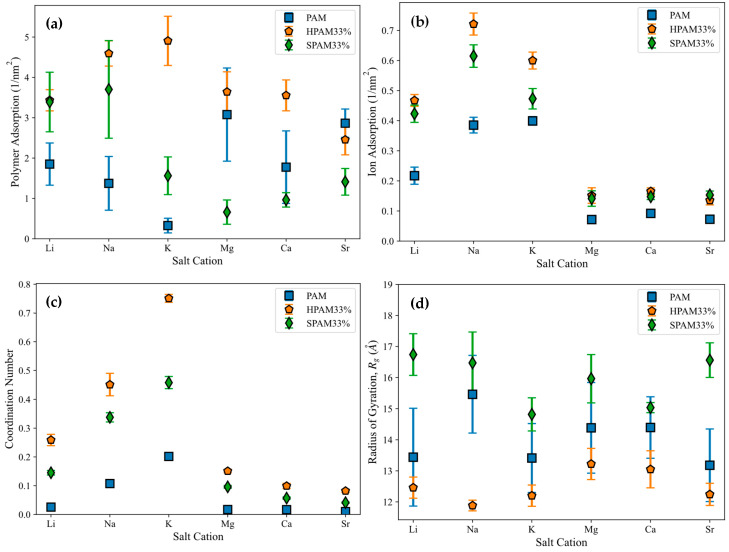
Simulation results of polymer adsorption with different salt cations: (**a**) Polymer adsorption to calcite surface, (**b**) cation adsorption to calcite surface, (**c**) cation adsorption to polymer, (**d**) polymer $R_{g}$. Polymer or cation adsorption to calcite surface are defined as the number of polymer chain atoms or cation atoms normalized by the surface area, counted within a layer thickness of 4 Å above the surface. Cation adsorption to polymer is defined as coordination number of cations with respect to carbonyl oxygen atom of polymer amide group (C(=O)NH_2_) in radial distribution function analysis. Blue square, orange pentagon, and green diamond indicate PAM, HPAM 33%, and SPAM 33%, respectively, with error bars representing standard error from 5 realizations.

In this section, the adsorption of various PAM-based polymers, including HPAM 33%, SPAM 33%, and neutral PAM, are investigated under varying cation valencies and ion sizes to observe the effect of their functional groups in saline environments, as presented in Figure 3a. The cation adsorption density on the surface is analysed similarly to polymer adsorption, with results displayed in Figure 3b. Additionally, radial distribution analysis is conducted to examine the adsorption of cations onto the polymer, as shown in Figure 3c. This is computed via the coordination number of the cations with respect to the carbonyl oxygen atom of the polymer amide group (C(=O)NH_2_). Lastly, the polymer conformational size is analysed using $R_{g}$ in Figure 3d.

The polymer adsorption results show that HPAM generally performs better than SPAM 33% and neutral PAM, with relatively high adsorption amounts measured across all cation cases. As explained in the previous adsorption free energy analysis, this can be attributed to the presence of carboxyl functional groups in HPAM, which experience stronger electrostatic attraction with the calcite surface compared to the bulkier functional groups in SPAM and the neutral ionicity of PAM. When comparing monovalent cations (Li^+^, Na^+^, K^+^) and divalent cations (Mg^2+^, Ca^2+^, Sr^2+^), interesting trends emerge among different polymers. HPAM adsorption is higher in monovalent cation cases than in divalent cation cases, with increasing monovalent cation sizes leading to increased adsorption, while increasing divalent cation sizes result in decreased adsorption. SPAM also shows reduced adsorption in divalent cation cases compared to monovalent cations. However, unlike HPAM, increasing monovalent cation sizes leads to decreased adsorption, while increasing divalent cation sizes results in increased adsorption. On the other hand, PAM adsorption is less influenced by cations. PAM shows better adsorption in divalent cation cases than in monovalent cation cases, with increasing monovalent cation sizes resulting in decreased adsorption and no significant trend observed for increasing divalent cation sizes.

**Figure 4:**
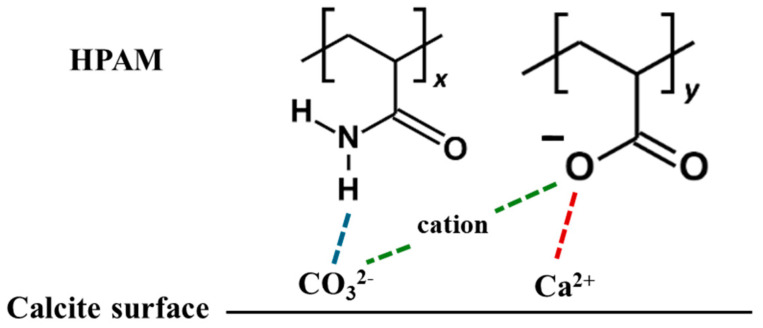
Schematic diagram of possible adsorption mechanism. **Blue bond**: hydrogen bonding between carbonate ion and amide group; **red bond**: charge interaction between calcium ion and carboxyl group; and **green bond**: salt bridging between carbonate anion and carboxyl group.

The differing adsorption behaviours among various polymers can be attributed to their distinct adsorption mechanisms influenced by cations. In the literature, the roles of cations in enhancing or inhibiting polymer adsorption have yielded contradictory results, with different mechanisms proposed to explain these behaviours. For polymer adsorption enhancement, some authors suggest that salt ions can form an Electric Double Layer (EDL) on the surface, facilitating salt bridging that attracts the polymer to the surface regardless of the polymer’s ionicities [17,23]. Conversely, polymer inhibition is attributed to the charge-screening effect of ions, which suppresses the polymer charge or causes surface charge reversal, thereby reducing the polymer’s adsorption strength to the surface [11,35]. In a saline environment, the adsorption of PAM-based polymers onto the calcite surface, focusing on HPAM, can be illustrated with the schematic diagram in Figure 4, with an electrostatic surface potential diagram provided as Figure S2 in the Supplementary Information.

Effective polymer adsorption to the calcite surface results from the competing effects of salt bridging and charge screening. In experiments, the calcite surface is positively charged under normal reservoir pH conditions [47], where cations may weaken the charge of an anionic polymer and screen its attraction to the surface. However, the calcite surface in the simulation is neutral, consisting of alternating CO_3_^2−^ and Ca^2+^ ions. HPAM can form hydrogen bonds with calcite CO_3_^2−^ via hydrogen atoms from the amide groups or significantly stronger charge interactions between calcite Ca^2+^ and deprotonated oxygen atoms from the carboxyl groups. While cations may neutralise the charge attraction between Ca^2+^ and deprotonated carboxyl groups, they can also function as salt bridges between CO_3_^2−^ and carboxyl groups, enhancing adsorption.

Simulation results suggest that salt bridging is more dominant in HPAM adsorption. HPAM’s higher charge density makes it more susceptible to cation influence than SPAM and PAM [31]. This claim agrees with the density profile analysis of the relevant oxygen atoms of the polymers and the cation (using Na^+^ as an example) during adsorption, as shown in Figure S3 in the Supplementary Information. This enhances the role of salt bridging, contributing to its higher adsorption across various cation cases. The differing adsorption trends between monovalent and divalent cations can be attributed to the hydration energy of the cations. Cations with stronger charge density form a more robust hydrogen-bonding network with water, exhibiting stronger hydration energy and preferring to remain in the bulk water phase. Such cations are known as water structure makers, while those with weaker hydration energy are termed water structure breakers [23].

Divalent cations exhibit significantly higher charge density than monovalent cations, while increasing ion sizes within the same valency can reduce charge density. Consequently, the hydration energy follows the following trend: Mg^2+^ > Ca^2+^ > Sr^2+^ > Li^+^ > Na^+^ > K^+^ [48]. This trend is reflected in the cation adsorption density shown in Figure 3b, where divalent cations, with stronger hydration energy, exhibit lower surface adsorption and prefer to remain in the aqueous phase compared to monovalent cations. The trend observed for monovalent cations also aligns with the hydration energy trend. In HPAM and SPAM cases, the lower adsorption of K^+^ compared to Na^+^ may be attributed to its larger ionic radius, which positions the adsorption peak further from the surface and may extend beyond the defined 4 Å adsorption layer [49].

The variation in cation adsorption density on the surface across different polymers indicates the influence of the polymer on cation adsorption. On the other hand, cation adsorption to the surface also affects the density of cations adsorbed onto the polymer. Higher cation adsorption to the surface creates a more concentrated salt layer, which attracts the polymer more effectively and increases the cation adsorption onto the polymer. As shown in Figure 3c, the cation adsorption onto the polymer exhibits a trend similar to that in Figure 3b, highlighting the interplay between cations and polymers. Consequently, these two factors contribute to the adsorption trends observed for different polymers under varying cation conditions depicted in Figure 3a.

**Figure 5:**
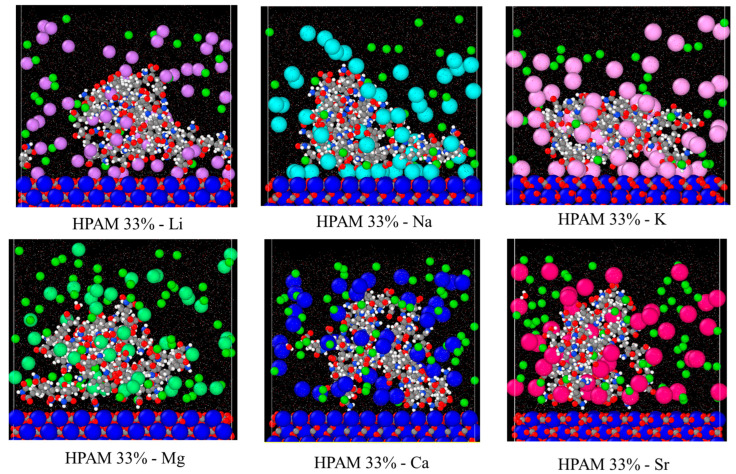
Simulation snapshot of HPAM 33% adsorption with different salt cations. Smaller particle—anions: Cl^−^ (forest green). Larger particle—cations: Li^+^ (purple), Na^+^ (cyan), K^+^ (pink), Mg^2+^ (mint green), Ca^2+^ (dark blue), Sr^2+^ (magenta).

Since the adsorption of HPAM to the surface is primarily driven by salt bridging, the observed increase in adsorption with larger monovalent cations is due to stronger cation adsorption onto the polymer (K^+^ > Na^+^ > Li^+^). Conversely, the decrease in adsorption with larger divalent cations is attributed to the reduced cation adsorption density on the polymer (Mg^2+^ > Ca^2+^ > Sr^2+^). The simulation snapshots in Figure 5 further illustrate this. Closer inspection reveals that a stable EDL is formed for monovalent cations, with cations creating the Stern layer (the first EDL layer) and Cl^−^ anions constituting the diffuse layer (the second EDL layer), stabilising the adsorption. In contrast, no stable EDL exists for divalent cations, resulting in lower adsorption. This observation aligns with previously reported cation interfacial interaction behaviours in calcite–water systems [49].

On the other hand, the differing trends observed for SPAM may be attributed to its lower charge strength, where cations have a reduced influence on polymer adsorption [31] or might even inhibit it through charge-screening effects. Compared to monovalent ions, SPAM exhibits lower adsorption to a calcite surface in divalent ions due to the stronger charge-screening effects. The decreasing adsorption trend with increasing monovalent cation size is attributed to stronger cation adsorption and greater charge screening. Conversely, the increasing adsorption trend with larger divalent cations results from a relatively lower charge-screening effect combined with reduced cation adsorption density.

Meanwhile, PAM, which has a neutral charge, primarily adsorbs onto the calcite surface through hydrogen bonding from amide groups, with adsorption governed mainly by surface oxygen densities [29]. Cations can inhibit this adsorption by screening the interaction between hydrogen atoms and calcite CO_3_^2−^. Consequently, the lower cation adsorption density on both the polymer and surface for divalent cations leads to higher PAM adsorption than monovalent cations. The decreasing trend in adsorption with increasing monovalent cation size is due to increasing cation adsorption density, resulting in more pronounced charge-screening effects. While cations inhibit adsorption in the case of PAM, they function as salt bridges between the anionic polymer and calcite CO_3_^2−^. For neutral PAM, anions may thus play a more dominant role in bridging interactions between the hydrogen atoms of the amide group and Ca^2+^. Although not presented here, a published study investigated the role of anions, showing that nitrate ions (NO_3_^−^) with a smaller hydrated radius and hydration energy than Cl^−^ can enhance the adsorption of neutral PAM on the calcite surface [50].

As illustrated in Figure 3d, polymer $R_{g}$ shows no significant trend across different cation cases. Among the polymers, SPAM has the largest size, followed by PAM and HPAM. Since all polymers have the same degree of polymerization, SPAM naturally exhibits the largest size due to its bulkier functional groups. Conversely, HPAM, with the smallest $R_{g}$, suggests a more compact structure. This is expected, as the cations strongly adsorb onto HPAM, neutralising its charge and causing the polymer to adopt a more compact conformation.

We conclude this section by comparing the experimental and simulation results for HPAM adsorption in salt-free conditions (control), NaCl, and CaCl_2_ environments, as presented in Table 6. Both experimental and simulation results show a similar trend for salt-free and Na^+^ cases, indicating that salt cations can enhance polymer adsorption through salt bridging. However, for divalent Ca^2+^, the simulation shows lower adsorption compared to Na^+^, while experiments reveal significantly higher adsorption. This discrepancy may be attributed to different interpretations of polymer adsorption. In experiments, Ca^2+^ provided more charges to neutralise the polymer, causing it to adopt a more coiled conformation and enabling more polymer chains to adsorb onto calcite particles, thereby increasing overall adsorption. In contrast, since only a single polymer chain is modelled in the simulation, the effects of multiple polymer chains adsorbing to the surface cannot be captured, leading to different observations.

## 3. Methodologies

### 3.1. General Simulation Details

The simulation details outlined here were applied uniformly across all MD simulations discussed in the previous sections. Classical atomistic molecular dynamics (MD) simulation was employed using Material Exploration and Design Analysis (Medea) simulation software v.3.5 [51] integrated with the LAMMPS module [52] with a built-in visualisation interface. All the molecules were described by the enhanced version of the all-atom Polymer Consistent Force Field (PCFF+) actively developed by MedeA (see Section S1 in Supplementary Information and ref. [6] for more forcefield parameter details and validation in our previous work), with a forcefield cut-off distance of 9.5 Å and the treatment of long-range electrostatic interaction with the particle–particle–particle Mesh (PPPM) method [53]. System minimisation was performed using the conjugate gradient method, and the simulations were conducted with the Velocity Verlet algorithm with a timestep of 1 fs. Periodic boundary conditions were applied in all Cartesian directions. Whenever an isobaric-isothermal ensemble (NPT) or a canonical ensemble (NVT) was performed, the system was controlled using the Nose–Hoover thermostat and barostat with the correction terms of Martyna, Tuckerman, and Klein [52,54] included in the equations of motion.

### 3.2. Calcite Model

The calcite structure was modelled to represent the carbonate rock. The unit cell was a rhombohedral crystal structure with a space group of *R*$\bar{3}$c. It had a dimension of *a* = *b* = 4.980 Å, *c* = 17.192 Å, and a plane angle of α = β = 90°, γ = 120°. It was further cleaved into a crystal plane (1 0 4), which has proven to be the most thermodynamically stable structure and has the lowest surface free energy, as validated in our previous work [6]. The crystal plane was replicated as a 6-layer thickness structure with 720 CaCO_3_ molecules and dimensions of *a* = 48.86 Å, *b* = 49.76 Å and *c* = 17.84 Å. This calcite structure was utilised across all the parametric studies.

### 3.3. Polymer Model

HPAM 33%, the polymer with the best adsorption performance from our previous work [6], was selected as the basis polymer model in the parametric studies. However, depending on the parameter of interest, other PAM-based polymers, such as basic PAM and SPAM 33%, were also modelled as polymer candidates. The oligomer repeat unit composition and copolymer ratio have been illustrated and detailed in Table 7. The “repeat unit” refers to the smallest representative structure forming a copolymerised PAM with a specific copolymer ratio. In the cases of HPAM 33% and SPAM 33%, the repeat unit comprised two acrylamide monomers and a deprotonated copolymer group, resulting in a structure with 33% charge density. In contrast, the corresponding size of the repeat unit of pure PAM consisted of three acrylamide monomers.

### 3.4. System Preparation and Configuration

**Figure 6:**
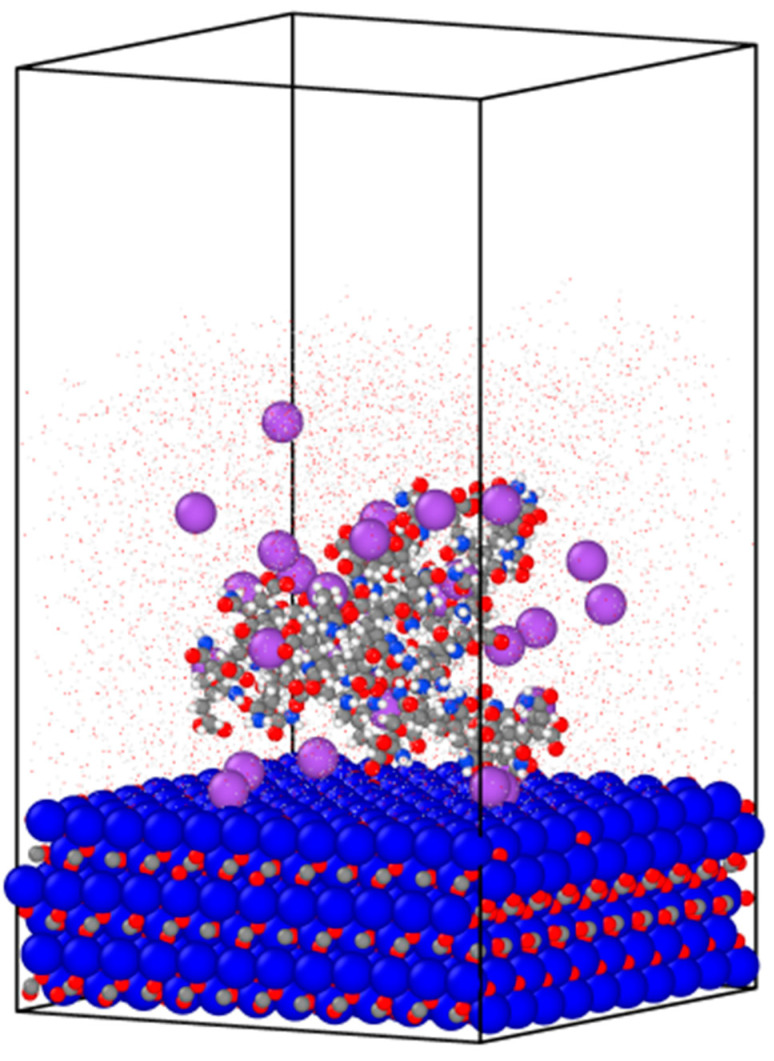
Initial configuration of the calcite–water system with HPAM 33% consisting of 30 repeat units. The sizes of the water molecules are reduced to tiny red dots for better visualisation. The remaining atoms are visualised with relative atom sizes and coloured based on element type. Calcite: Ca^2+^ (deep blue), carbon (grey), oxygen (red). HPAM: carbon (grey), oxygen (red), hydrogen (white), nitrogen (light blue). Na^+^ (purple).

For the aqueous environment, 3000 water molecules were placed above the calcite surface, with a single polymer chain placed within the water solvent. HPAM 33%, the basis polymer model, consisted of 30 repeat units. To ensure electroneutrality in the simulation environment, 30 sodium ions were randomly placed within the solvent. A vacuum layer was added to the simulation box to isolate one of the calcite surfaces and direct the adsorption of the polymer onto a single surface layer. This setup prevents the competitive adsorption of the polymer to both surfaces during simulation, allowing for more consistent results analysis. The resulting simulation system had dimensions of *a* = 48.86 Å, *b* = 49.76 Å, and *c* = 86.38 Å, as illustrated in Figure 6.

Table 8 includes the designated parameter conditions and system descriptions for different parameters. In general, the simulation study for each parameter considered the lower, intermediate, and higher ranges to compare with experimental results. When varying a specific parameter, other parameters were kept constant at the control condition of HPAM 33% with 30 repeat units at 25 °C in a salt-free environment. All the experimental candidates were HPAM polymers, with labels representing different polymer chemistries, as detailed in the published work [55].

When examining polymer chemistries such as polymer molecular weight and charge density, the HPAM structure was adjusted accordingly to mimic experimental candidates. For example, HPAM 33% with 10, 20, and 30 repeat units were constructed when investigating polymer molecular weight. In terms of polymer charge density, the polymer repeat unit was modified to match the charge density of the experimental candidates. HPAM chains with 10% (repeat unit consisting of nine acrylamide monomers and one acrylate monomer), 30% (repeat unit composed of seven acrylamide monomers and three acrylate monomers), and 40% (repeat unit consisting of six acrylamide monomers and four acrylate monomers) charge densities were constructed. Each chain had six repeat units, with the negative charges distributed evenly across the chains.

In some parameter conditions, such as high-pressure scenarios that are challenging to achieve experimentally, simulation results were analysed independently to provide predictive insights into the adsorption behaviours. Similarly, when assessing the effect of salinity, where experimental data are limited to NaCl and CaCl_2_, simulations can explore the impact of salt ion valency with varying ion sizes on the adsorption mechanism. Three monovalent cations, lithium (Li^+^), sodium (Na^+^), and potassium (K^+^), and three divalent cations, magnesium (Mg^2+^), calcium (Ca^2+^), strontium (Sr^2+^), were modelled in this study, all paired with chloride anions (Cl^−^). To recreate the three wt% NaCl solution in experiments, 29 NaCl molecules, equivalent to 0.53M, were placed in the solvent. The same number of cations and anions were placed for different cation cases to ensure consistency, with double the number of anions for divalent cations (MgCl_2_, CaCl_2_ and SrCl_2_). Additionally, neutral PAM and SPAM 33% were investigated together with HPAM 33% in this parameter study, all with 30 repeat units, to observe the effect of polymer functional groups in a saline environment. The counterions placed in HPAM 33% and SPAM 33% systems consisted of the corresponding cations (30 and 15 counterions for monovalent and divalent cations, respectively).

### 3.5. Simulation Details and Analysis Methods

The system was equilibrated for each parameter using the NVT ensemble at 300 K for 500 ps, followed by a production run of 30 ns under the same simulation conditions. The only exception was during the investigation of pressure, where the simulation was performed under the NPT ensemble to adjust the pressure accordingly. The polymer adsorption behaviour was observed, and the adsorption amount on the calcite was computed. This adsorption amount, or adsorption surface density, was defined as the number of polymer chain atoms bound within a layer thickness of 4 Å above the calcite surface. The total number of adsorbed polymer atoms was then normalized by the calcite surface area.

**Figure 7:**
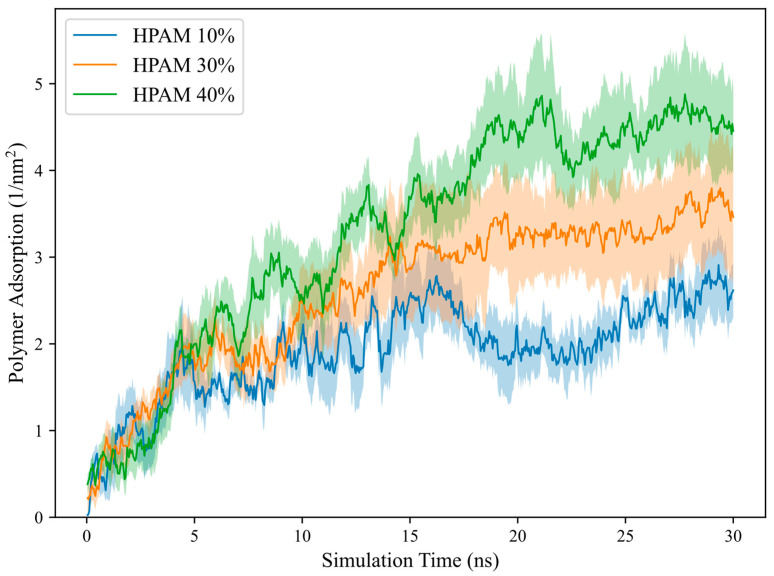
Polymer adsorption time profile for polymer charge density case. HPAM 10%, 30%, and 40% refer to polymers with charge densities of 10%, 30% and 40%, respectively. Shaded areas represent standard error from 5 realizations.

We highlight that the simulation time during the production run was sufficient for polymer equilibration for qualitative analysis, as shown by the adsorption time profile in Figure 7 for the polymer charge density parameter. Only the averaged polymer adsorption amount was reported in the results section, averaged over the last 2 ns across five different realizations and compared with experimental results whenever available. As the adsorption simulations require extremely long times to reach true equilibrium, this relatively short sampling interval of 2 ns is chosen to minimize fluctuations and provides more consistent computed values. It is also noted that during the preliminary study, the results were not affected by the system size effect, as the adsorbed amount of polymer with 30 repeat units showed no significant change when placed into a larger calcite–water system. Furthermore, some water molecules could evaporate to the opposite surface under the vacuum slab during the simulation, forming a thin, stabilized water layer. Nevertheless, the number of water molecules evaporated to the surface slab was negligible and did not affect polymer adsorption density.

For the experimental methodologies, the polymer adsorption measurement workflow has been detailed in published works [6,39] and will not be reiterated here. The experimental adsorption data were taken from the equilibrium adsorbed value at the optimum polymer concentration [56]. Due to the differences in the scales and methodologies in quantifying polymer adsorption, it is important to note that the comparison between simulation and experimental results is purely qualitative with trend indications only.

The polymer conformation behaviour during the adsorption can be assessed across different parameters. Although the radius of gyration, $R_{g}$ can be employed to measure the polymer size, it does not provide meaningful analysis of the polymer shape, such as whether they adopt an extended or coiled conformation. While the polymer shape may be interpreted as a flat, extended conformation with a larger $R_{g}$ or a compact, coiled conformation with a smaller $R_{g}$ under similar polymer structures, such analysis becomes difficult when assessing polymers of different sizes. A more useful property to characterise the shape is the relative shape anisotropy, $\kappa^{2}$. $\kappa^{2}$ can be computed from the gyration of tensor, S, adapted from [57]:

(7) $$S=\frac{1}{N}\left(\begin{array}{lll} \sum_{i}{\left(x_{i}-x_{C}\right)}^{2} & \sum_{i}\left(x_{i}-x_{C}\right)\left(y_{i}-y_{C}\right) & \sum_{i}\left(x_{i}-x_{C}\right)\left(z_{i}-z_{C}\right)\\ \sum_{i}\left(y_{i}-y_{C}\right)\left(x_{i}-x_{C}\right) & \sum_{i}{\left(y_{i}-y_{C}\right)}^{2} & \sum_{i}\left(y_{i}-y_{C}\right)\left(z_{i}-z_{C}\right)\\ \sum_{i}\left(z_{i}-z_{C}\right)\left(x_{i}-x_{C}\right) & \sum_{i}\left(z_{i}-z_{C}\right)\left(y_{i}-y_{C}\right) & \sum_{i}{\left(z_{i}-z_{C}\right)}^{2} \end{array}\right)\;$$

where subscript i represents atom i, C represents the centre of mass, and x, y, and z indicate the atomic coordinate in three directions, respectively. The eigenvalues of the matrix can be obtained by the diagonalization of S and are commonly sorted in descending order ($\lambda_{1}$>$\lambda_{2}$ >$\lambda_{3}$).

(8) $$S=diag(\lambda_{1},\lambda_{2},\lambda_{3})$$

$R_{g}$ and $\kappa^{2}$ can then be expressed as:

(9) $$R_{g}^{2}=\lambda_{1}+\lambda_{2}+\lambda_{3}$$

(10) $$\kappa^{2}=1-3\frac{\left(\lambda_{1}\lambda_{2}+\lambda_{2}\lambda_{3}+\lambda_{3}\lambda_{1}\right)}{{\left(\lambda_{1}+\lambda_{2}+\lambda_{3}\right)}^{2}}$$

$\kappa^{2}$ essentially characterises the dimensionality and symmetry of the polymer, ranging from 0 to 1. A value of 0 signifies a perfectly spherical shape, indicating a more coiled polymer conformation, while a value of 1 denotes a linear chain, reflecting a more extended conformation. It is noted that these terms are typically employed to describe a free polymer in solution. During polymer adsorption, the surface restricts the polymer’s movement and distribution, effectively reducing its conformation freedom and causing it to exist in quasi-two-dimensional space. In the current study, the adsorbed polymer conformation may still be described with Equations (9) and (10), but with a slightly different interpretation.

## 4. Conclusions

This study has employed molecular dynamics simulations to investigate the adsorption behaviour of hydrolysed polyacrylamide (HPAM) onto calcite surfaces under various reservoir conditions. Our findings demonstrate that HPAM adsorption is enhanced by higher molecular weight, charge density, and temperature, while pressure exerts minimal influence. The interplay between salt-bridging and charge-screening mechanisms governs the impact of salinity on adsorption, with salt bridging being more pronounced for HPAM. While the simulation results generally correlate with experimental observations, discrepancies may arise from limitations in the simulation model and forcefield [58]. Overall, these findings provide valuable insights into the design and optimisation of polymer-based solutions for solids production control in carbonate reservoirs.

## Figures and Tables

**Table 1:** Polymer adsorption comparison between experiments and MD simulations, $R_{g}$ and $\kappa^{2}$ at different molecular weights.

HPAM Repeat Units	Experimental Adsorbed Amount (mg/m^2^)	MD Adsorbed Amount (1/nm^2^)	$R_{g}$ (Å)	$\kappa^{2}$
10	0.250 ± 0.014	2.731 ± 0.575	9.48 ± 1.21	0.474 ± 0.111
20	0.330 ± 0.020	3.476 ± 0.370	10.27 ± 0.38	0.211 ± 0.068
30	0.420 ± 0.010	4.370 ± 0.126	11.97 ± 0.27	0.126 ± 0.032

**Table 2:** Polymer adsorption comparison between experiments and MD simulations, $R_{g}$ and $\kappa^{2}$ at different charge densities.

HPAM Charge Density (%)	Experimental Adsorbed Amount (mg/m^2^)	MD Adsorbed Amount (1/nm^2^)	$R_{g}$ (Å)	$\kappa^{2}$
10	0.160 ± 0.008	2.634 ± 0.386	10.01 ± 0.19	0.151 ± 0.028
30	0.240 ± 0.014	3.596 ± 0.649	11.55 ± 0.93	0.276 ± 0.095
40	0.265 ± 0.011	4.580 ± 0.563	13.41 ± 1.33	0.370 ± 0.094

**Table 3:** Comparison of polymer adsorption between experiments and simulations at different temperatures.

System Temperature (°C)	Experimental Adsorbed Amount (mg/m^2^)	MD Adsorbed Amount (1/nm^2^)
25	0.240 ± 0.014	4.370 ± 0.126
60	0.190 ± 0.018	6.642 ± 1.158
100	0.110 ± 0.011	9.052 ± 0.915

**Table 4:** Polymer $R_{g}$, $\kappa^{2}$, and D at different temperatures.

System Temperature (°C)	$R_{g}$ (Å)	$\kappa^{2}$	D (10^−11^ m^2^/s)
25	11.97 ± 0.27	0.126 ± 0.032	4.30 ± 1.08
60	12.51 ± 0.36	0.132 ± 0.024	5.88 ± 1.39
100	14.19 ± 0.95	0.268 ± 0.068	13.85 ± 3.71

**Table 5:** Polymer adsorption, $R_{g}$, and $\kappa^{2}$ at different pressures.

System Pressure (atm)	MD Adsorbed Amount (1/nm^2^)	$R_{g}$ (Å)	$\kappa^{2}$
1	4.525 ± 0.561	11.91 ± 0.25	0.123 ± 0.023
80	5.438 ± 0.385	11.91 ± 0.23	0.106 ± 0.019
160	5.237 ± 0.181	12.06 ± 0.36	0.123 ± 0.022

**Table 6:** Comparison of polymer adsorption between experiments and simulations at salt cation valencies.

Salt Cation Valency	Experimental Adsorbed Amount (mg/m^2^)	MD Adsorbed Amount (1/nm^2^)
Salt-free condition	0.330 ± 0.020	4.370 ± 0.126
Na	0.380 ± 0.036	4.593 ± 0.313
Ca	0.820 ± 0.137	3.554 ± 0.383

**Table 7:** Polymer compositions and molecular weight. *x* and *y* refer to the ratio of copolymers in a repeat unit.

Polymer	Repeat Unit Composition	Copolymer Ratio	Molecular Weight per Repeat Unit (Da)
PAM	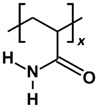	*x* = 3 (repeat unit consists of 3 acrylamide monomers)	213.91
HPAM 33%	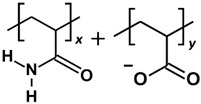	*x:y =* 2:1	213.89
SPAM 33%	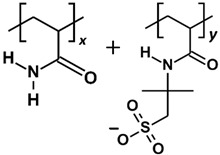	*x:y =* 2:1	372.06

**Table 8:** Designated parameter spaces and system descriptions for adsorption studies with experimental conditions. Control system details: HPAM polymer with 30 repeat units and 33% charge density at 25 °C in salt-free conditions.

Parameter	System Description		MD Simulation	Experimental Condition
Polymer molecular weight (MW)	HPAM repeat units (213 Da/repeat unit)	Lower MW	10	F3330S (11–13 MDa)
		Middle MW	20	F3530S (15–17 MDa)
		Higher MW	30	F3630S (18–20 MDa)
Polymer charge density (CD)	HPAM charge density (%)	Lower CD	10	AN910 (10%)
		Middle CD	30	F3330S (30%)
		Higher CD	40	AN945 (40%)
Temperature (T)	System temperature (°C)	Lower T	25	F3330S, 25
		Middle T	60	F3330S, 80
		Higher T	100	F3330S, 95
Pressure (P)	System pressure (atm)	Lower P	1	-
		Middle P	80	-
		Higher P	160	-
Salinity	Saline environment with different cations but same anions, Cl⁻	Monovalent cation	Li^+^	-
		Monovalent cation	Na^+^	F3530S, 3 wt% NaCl
		Monovalent cation	K^+^	-
		Divalent cation	Mg^2+^	-
		Divalent cation	Ca^2+^	F3530S, 3 wt% CaCl_2_
		Divalent cation	Sr^2+^	-

## Data Availability

Data supporting this manuscript, including example input files to perform equilibrium adsorption across different parameters can be downloaded at Figshare (DOI://10.6084/m9.figshare.27890982).

## Supplementary Materials

The following supporting information can be downloaded at: https://www.mdpi.com/article/10.3390/molecules30020285/s1, S1. Forcefield Structure and Parameters Info, S2. Electrostatic Surface Potential Diagram for Possible Polymer-Calcite Adsorption Mechanism, S3. Density Profile Distribution of Cation and Polymer Atoms During Adsorption on Calcite Surface.
