# Supplementary material for: Parametric Studies of Polyacrylamide Adsorption on Calcite Using Molecular Dynamics Simulation
Source: Molecules. 2025 Jan 13;30(2):285. doi: 10.3390/molecules30020285 (PMC11767760; doi:10.3390/molecules30020285)
Supplement: Supplementary file 1 [file molecules-30-00285-s001.zip › molecules-3359115-supplementary.pdf]

Parametric Studies of Polyacrylamide Adsorption on Calcite Using Molecular Dynamics Simulation Supplementary Information

Keat Yung Hue[1], Jin Hau Lew[1], Omar K. Matar[1], Paul F. Luckham[1], Erich A. Müller[1]*
[1]Department of Chemical Engineering, Imperial College London, London SW7 2AZ, UK,
*Corresponding author e-mail: e.muller@imperial.ac.uk

S1. Forcefield Structure and Parameters Info

For PCFF+ forcefield, the non-bonded terms consist of Mie 9-6 potential and charged Coulombic electrostatic interactions as shown in **Equation S1**. For different atom types, the sixth power combining rule in **Equation S2** is used to compute the unlike binary interaction parameters.

$$E_{non-bonded} = \varepsilon\left[2\left(\frac{\sigma}{r}\right)^9 - 3\left(\frac{\sigma}{r}\right)^6\right] + \alpha\frac{Cq_iq_j}{\epsilon r} \qquad \textbf{(S1)}$$

$$\sigma_{ij} = \left(\frac{1}{2}\left(\sigma_i^6 + \sigma_j^6\right)\right)^{\frac{1}{6}} \qquad \varepsilon_{ij} = \frac{2\sqrt{\varepsilon_i\varepsilon_j}\sigma_i^3\sigma_j^3}{\sigma_i^6 + \sigma_j^6} \qquad \textbf{(S2)}$$

where ε is potential well depth at minimum potential energy, σ is a characteristic segment distance at which the potential energy is zero, r is interatomic distance between atom i and atom j, α is damping factor, C is energy conversion constant, q is the charge of the atom and ϵ is dielectric constant.

A list of the parameters for the molecule involved in our study are included in **Table S1**, with the designation of the atoms type labelled depicted in **Figure S1**. As the intra-molecular potential parameters are rather complicated, it will not be listed here but are available upon request.

Table S1. Non-bonded parameters of different molecules assigned with PCFF+.

Molecule	Atom Type Element	Atom Type label	ε (kcal/mol)	σ (Å)
**PAM**	Carbon	c1	0.0350	3.7500
	Carbon	c2	0.0634	3.7584
	Carbon	c3	0.0710	3.8840
	Carbon	c_1	0.0640	3.9000
	Hydrogen	h	0.0230	2.8780
	Hydrogen	hn2	0.0120	1.4000
	Nitrogen	n_32	0.2314	3.9742

	Oxygen	o_1	0.1920	3.4300
**HPAM**	Carbon	c-	0.1200	3.9080
	Carbon	c1	0.0350	3.7500
	Carbon	c2	0.0634	3.7584
	Carbon	c3	0.0710	3.8840
	Carbon	c_1	0.0640	3.9000
	Hydrogen	h	0.0230	2.8780
	Hydrogen	hn2	0.0120	1.4000
	Nitrogen	n_32	0.2314	3.9742
	Oxygen	o-	0.1670	3.5960
	Oxygen	o_1	0.1920	3.4300
**SPAM**	Carbon	c0	0.0070	3.7500
	Carbon	c1	0.0350	3.7500
	Carbon	c2	0.0634	3.7584
	Carbon	c3	0.0710	3.8840
	Carbon	c_1	0.0640	3.9000
	Hydrogen	h	0.0230	2.8780
	Hydrogen	hn2	0.0120	1.4000
	Nitrogen	n_31	0.2000	3.9200
	Nitrogen	n_32	0.2314	3.9742
	Oxygen	o1s-	0.1960	3.6400
	Oxygen	o_1	0.1920	3.4300
	Sulphur	sf-	0.2400	4.0800
**Calcite**	Carbon	c3o-	0.0634	3.7584
	Calcium	ca+	0.3404	3.2990
	Oxygen	o1c-	0.1960	3.6400
**Water**	Hydrogen	hw	0.0130	1.0980
	Oxygen	o*	0.0700	3.8150
**Cations & Anion**	Lithium	Li^+	0.3727	1.6350
	Sodium	Na^+	0.3828	2.4940
	Potassium	K^+	0.4691	3.2920
	Magnesium	Mg^{2+}	0.2951	2.3959
	Calcium	Ca^{2+}	0.3404	3.2990
	Strontium	Sr^{2+}	0.3363	3.6774
	Chloride	Cl^-	0.05899	5.1728

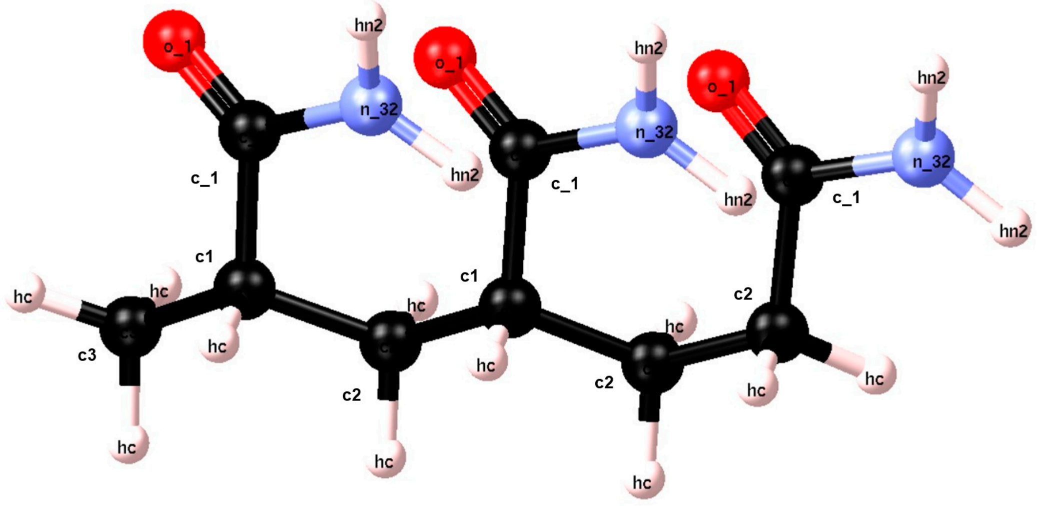

Figure S1. (a) Atom type label for a repeat unit of PAM.

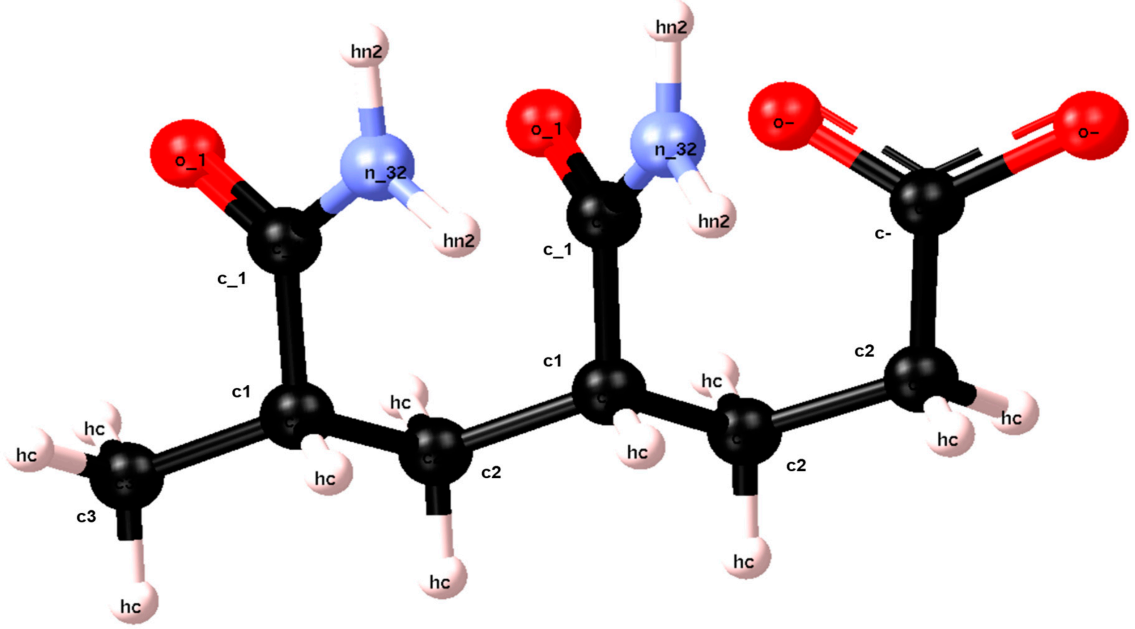

Figure S1. (b) Atom type label for a repeat unit of HPAM.

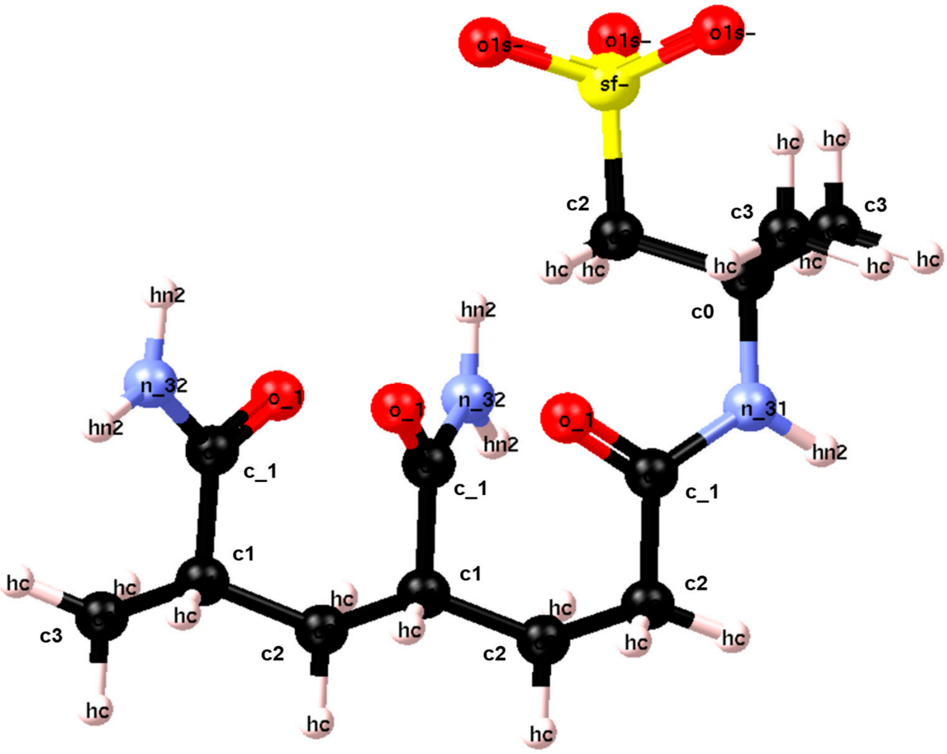

Figure S1. (c) Atom type label for a repeat unit of SPAM.

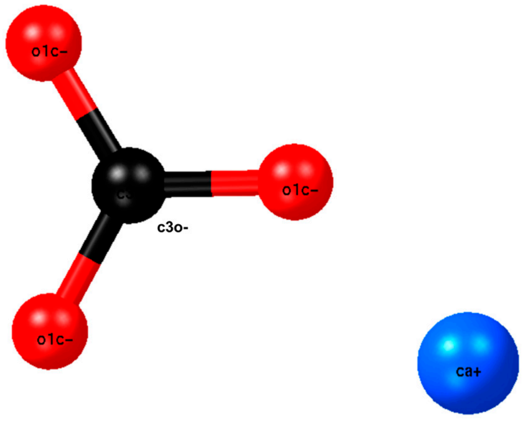

Figure S1. (d) Atom type label for a pair of calcium carbonate structure.

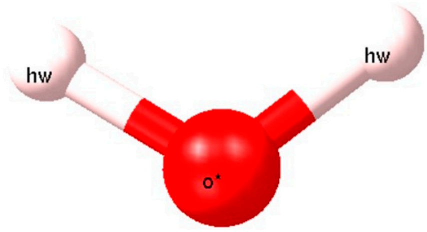

Figure S1. (e) Atom type label for a water molecule.

S2. Electrostatic Surface Potential Diagram for Possible Polymer-Calcite Adsorption Mechanism

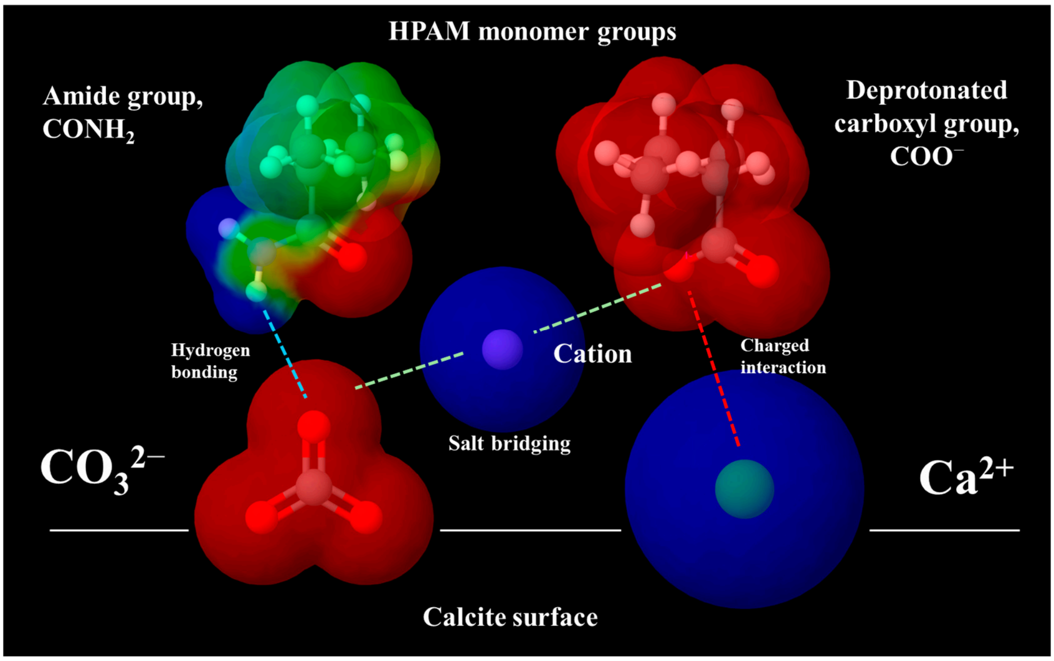

Figure S2: Electrostatic surface potential (ESP) diagram illustrating the possible polymer adsorption mechanism on the calcite surface in the presence of cations. The blue regions indicate

areas deficient in electrons (positively charged), while the red regions indicate areas rich in electrons (negatively charged). Green region represents neutral segments. See the schematic diagram shown in **Figure 6** in the main text for clearer depiction of atom label.

S3. Density Profile Distribution of Cation and Polymer Atoms During Adsorption on Calcite Surface

Figure S3 shows the atom number density profiles of the cation (using Na+ as an example) and the relevant oxygen atoms in the polymer during adsorption onto the calcite surface.

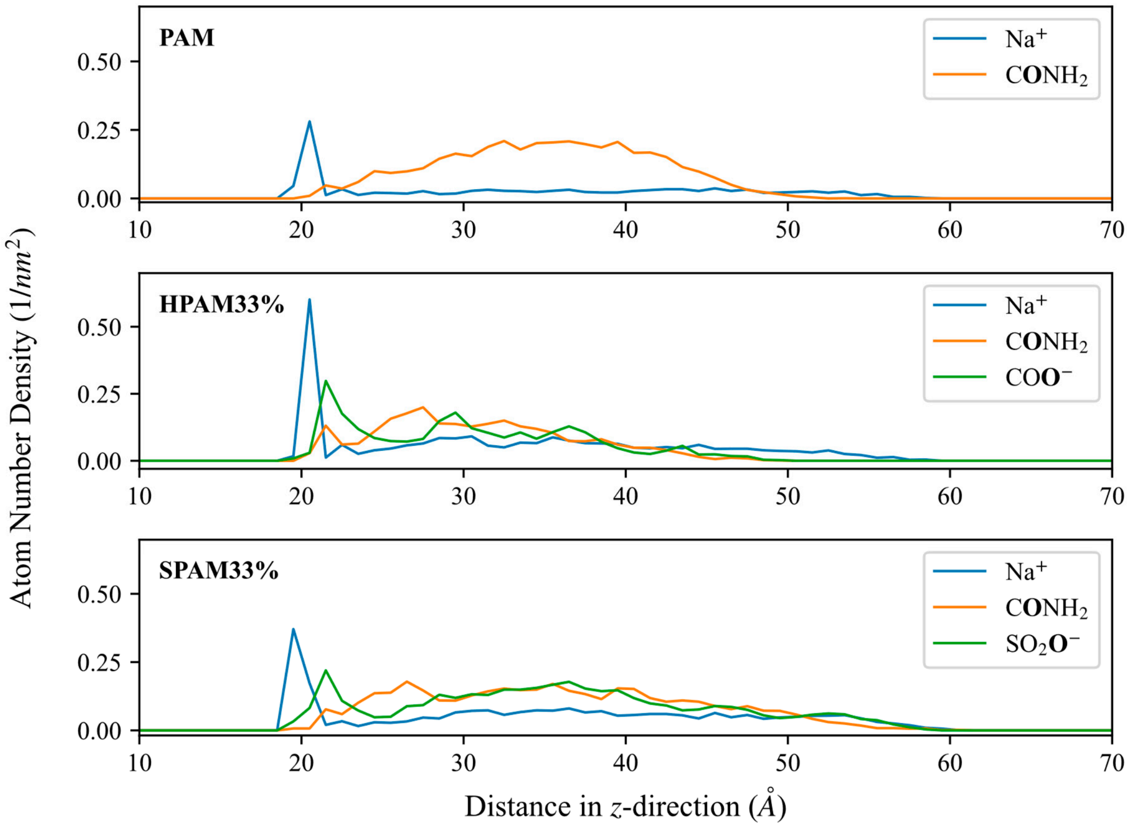

Figure S3: Atom number density profiles of the cation, Na+ and the relevant oxygen atoms in the polymer during adsorption onto the calcite surface upon reaching equilibrium. Distance near 20Å indicates the calcite slab surface location. **PAM, HPAM and SPAM cases**: blue line represents density profile of Na^+ while orange line represents density profile of oxygen atom from the amide group (C**O**NH_2). **HPAM**: green line represents density profile of charged oxygen atom from deprotonated carboxyl (CO**O**⁻) group. **SPAM**: green line represents density profile of charged oxygen atom from deprotonated sulfonate (SO_2**O**⁻) group.

For PAM, being a neutral polymer, only one type of oxygen atom is available: the oxygen from the amide group (C**O**NH_2). In contrast, HPAM and SPAM include additional ionized oxygen atoms due to their deprotonated carboxyl (CO**O**$^-$) and sulfonate (SO_2**O**$^-$) groups, respectively. It is important to note that more Na+ ions are present in the HPAM and SPAM cases due to the need for counterions to maintain electroneutrality. The results show that Na+ exhibits the highest peak density in the HPAM case compared to SPAM and PAM.

For all cases, the peak density of the oxygen atom from the C**O**NH_2 group is located further from the Na^+ peak density, indicating that the amide group is not dominantly attracted to and influenced by the cations. Meanwhile, the charged oxygen atom from the CO**O**$^-$ group in HPAM demonstrates a higher peak density and is closer to the Na^+ peak density compared to the SO_2**O**$^-$ group in SPAM. This is likely due to the bulkier nature of the sulfonate group in SPAM, which increases the distance between the peaks.

These observations align with the higher polymer adsorption amount observed in HPAM. The charged oxygen atoms in HPAM play a more dominant role by being strongly attracted to the salt cations, which, in turn, adsorb better to the calcite surface via the salt bridging effect. This quantitative analysis supports the proposed adsorption mechanism in the main text in **section 2.5**.
